# Biomonitoring of Aflatoxin B_1_ and Deoxynivalenol in a Rural Pakistan Population Using Ultra-Sensitive LC-MS/MS Method

**DOI:** 10.3390/toxins12090591

**Published:** 2020-09-12

**Authors:** Lei Xia, Michael N. Routledge, Hifza Rasheed, Amir Ismail, Yao Dong, Tao Jiang, Yun Yun Gong

**Affiliations:** 1 School of Food Science & Nutrition, University of Leeds, Leeds LS2 9JT, UK; fslx@leeds.ac.uk (L.X.); florady0327@163.com (Y.D.); s6171550@live.tees.ac.uk (T.J.); 2 School of Medicine, University of Leeds, Leeds LS2 9JT, UK; M.N.Routledge@leeds.ac.uk; 3 School of Food and Biological Engineering, Jiangsu University, Zhenjiang 212013, Jiangsu, China; 4 Pakistan Council of Research in Water Resources, Islamabad 44080, Pakistan; hifzajohar@gmail.com; 5 Institute of Food Science and Nutrition, Bahauddin Zakariya University, Multan 60000, Pakistan; amirismail@bzu.edu.pk

**Keywords:** aflatoxin, deoxynivalenol, exposure assessment, human biomonitoring, UPLC-MS/MS

## Abstract

There are limited data on exposure to mycotoxins in Pakistan. Here, we measured exposure to deoxynivalenol (DON), a common contaminant of wheat, and aflatoxin B_1_ (AFB_1_), a known contaminant of rice, using biomarkers of exposure. Wheat (*n* = 195) and rice (*n* = 62) samples were analyzed for AFB_1_ and DON levels, and the corresponding urinary biomarkers were analyzed in urine samples from a rural population (*n* = 264, aged 4–80 years, male 58%) using ultra-sensitive liquid chromatography–tandem mass spectrometry. AFB_1_ was detected in 66% of rice (5.04 ± 11.94 µg/kg) and 3% of wheat samples. AFM_1_ (hydroxylated form of AFB_1_) was detected in 69% of urine samples, mean 0.023 ± 0.048 ng/mL and DON was detected in 20% of urine samples, mean 0.170 ± 0.129 ng/mL. The maximum probable daily intake for DON derived from the urinary biomarker was 59.8 ng/kg b.w./day, which is below the Joint Food and Agriculture Organization/World Health Organization Expert Committee on Food Additives’ tolerable daily intake (1000 ng/kg b.w./day). However, for aflatoxin, the derived margin of exposure (MoE) of (13.2) was well below the safe MoE (10,000) suggested by the European Food Safety Authority. The calculated aflatoxin-associated cancer risk of 0.514/10^5^ individuals/year suggests that measures should be taken to reduce the AFB_1_ contamination in food, particularly rice, in Pakistan.

## 1. Introduction

Mycotoxins are toxic secondary metabolites produced by fungi that contaminate food crops at the pre- and/or post-harvest stages of cereals, oilseeds, spices, and nuts and the main exposure route is via dietary exposure [1,2]. Depending on the mycotoxin in question, toxicity can include genotoxicity, carcinogenicity, mutagenicity, teratogenicity, and immune-toxicity [3].

Aflatoxin B_1_ (AFB_1_) is the most potent and widely studied mycotoxin, principally produced by *Aspergillus flavus* and *Aspergillus parasiticus* that primarily contaminate the food category “legumes, nuts, and oilseeds” as well as cereals [4]. The AFB_1_ and aflatoxin natural mixture (AFB_1_, AFB_2_, AFG_1_, AFG_2_) are classified as Group 1 carcinogens by the International Agency for Research on Cancer [5]. Past studies have shown a strong association between increased risk of hepatocellular carcinoma and chronic AFB_1_ exposure [6]. High dose, short exposure causes liver damage and high rate of fatalities, e.g., in Tanzania in 2016 [7]. AFB_1_ has also been found to be associated with child growth impairment, suppression of immune function, and hepatomegaly [8,9,10,11,12]. Rice is a staple food in parts of Pakistan and can be susceptible to aflatoxin contamination.

Another staple crop in Pakistan is wheat, which is less susceptible to aflatoxin contamination but often contaminated with another mycotoxin, deoxynivalenol (DON), a type-B trichothecene produced by *Fusarium* fungi. Adverse effects of DON including feed refusal and slowed growth have been reported in animals [13]. The symptoms of acute DON exposure in humans are vomiting and other gastrointestinal symptoms, but the effect of chronic exposure to DON is not yet well understood. The Joint Food and Agriculture Organization/World Health Organization Expert Committee on Food Additives (JECFA) has set up a provisional maximum tolerable daily intake (PMTDI) of 1000 ng/kg b.w./day for the total amount of DON and its acetylated derivatives [14]. In Pakistan, evidence of DON contamination in food has been reported, but data are limited [15,16,17]. A recent study showed high levels of DON in 449 wheat and 270 corn samples collected from the studied area (more than 40% with mean level around 1000 µg/kg) [18]. 

Urinary AFM_1_ (hydroxylated form of AFB_1_) and AFB_1_-albumin adduct in blood are frequently used biomarkers of aflatoxin exposure [6]. For DON, urinary DON and its metabolites have been established to correlate well with dietary exposure [19,20]. 

The reports of mycotoxin contamination of food in Pakistan [17,21,22] highlight the need for a biomonitoring study in the Pakistan population. The present study aimed to assess exposure and risk associated with the intake of AFB_1_ and DON in rural Pakistan. 

## 2. Results

### 2.1. Analytical Method Validation

For both rice/wheat and urine samples, extraction and analysis were performed in batches. For the wheat and rice samples, each batch contained two quality controls (QC) spiked with the same level of AFB_1_ and DON. For the urine samples, each batch contains three quality controls (QC) spiked with different levels of AFM_1_ or DON. These quality controls were used to evaluate the recovery, reliability, and variation of the method. The 15 batches of rice/wheat QC samples showed a stable small coefficient of variance (CV) of 10% and good recovery (91% ± 8.8%) for the analysis, which suggests the analytical method was reliable and stable. The data for the 15 batches of urine QCs are summarized in Table 1. As shown in the table, both urinary AFM_1_/DON analysis also showed good recoveries (around the range of 80–120%) at spiked levels of concentration. The CV of the 15 sets of QCs illustrated a satisfactory variation of the method. A random sample of 10% of the total sample was extracted again to assess the reproducibility of the method, and no significant differences (*p* > 0.05) were observed between the original samples and repeated samples for both the rice/wheat AFB_1_/DON levels and urinary AFM_1_/DON levels.

### 2.2. Demographic Characteristics

Demographic data are summarized in Table 2. The average age of the participants with available urine samples (*n* = 264) was 35 ± 17 years (range 4–80 years). The locations of the six villages have been described in the previous study [23]. One-way ANOVA was used to test the difference in age between villages and participants from village 48 showed significantly higher age (*p* = 0.036). Village Chak-46 from Sahiwal had the highest number of participants (37.5%). Body weight was also obtained from the cohort, with an average of 55.7 ± 20.4 kg, but since “height” was not included in the questionnaire, it was not possible to calculate the body mass index (BMI) of the cohort. Weight was used to calculate wheat/rice consumption per kg body weight. The majority (70%) of the participants were farmers or housewives, who are also normally involved in farming practices. The majority (99.2%) of the participants consumed chapatti (one of the staple foods in Pakistan, made from wheat flour). On average, 41.3% had consumed rice the previous day. 

### 2.3. Exposure of Population to AFB_1_

Results for the AFB_1_ contamination level analysis in rice and wheat samples are summarized in Table 3. Overall, 66% of the collected rice samples showed a detectable level of AFB_1_, with a mean of 5.04 ± 11.94 µg/kg ranging from <LOD (limit of detection) to 71.56 µg/kg. Significantly different levels of AFB_1_ in rice were observed between villages (*p* = 0.04), among which the village Basti Balochan (BB) of Bahawalpur district showed the highest average AFB_1_ level in rice (10.17 ± 18.02 µg/kg). In contrast to rice, only 2% of the collected wheat samples showed a detectable level of AFB_1_ with a mean of 0.04 ± 0. 12 µg/kg ranging from <LOD to 1.59 µg/kg. 

Urinary AFM_1_ levels are summarized in Table 4. AFM_1_ was detectable in 69% of urine samples, with an average level of 0.023 ± 0.048 ng/mL ranging from <LOD to 0.393 ng/mL. Apart from village BB, all other villages had participants with urinary AFM_1_ level higher than 0.1 ng/mL. A significant difference (*p* < 0.001) was observed between the six villages among which village 46 and Badarpur (BP) showed the highest prevalence and average of urinary AFM_1_ level of 0.039 and 0.037 ng/mL, respectively.

The estimate dietary intake (EDI) and probable daily intake (PDI) of AFB_1_ can be derived from either the rice/wheat AFB_1_ contamination level using Equation (1) or from the urinary AFM_1_ level using Equation (2), respectively. The average EDI was estimated to be 3.5 ng/kg b.w./day and the average PDI was estimated to be 30.3 ng/kg b.w./day for AFB_1_.

### 2.4. Exposure of Population to DON

None of the wheat or rice samples showed detectable levels of DON. DON biomarker results are summarized in Table 4. Only 20% of the urine samples had detectable urinary DON, mean 0.170 ± 0.129 ng/mL, with a maximum value of 1.247 ng/mL. Following this, it was possible to calculate the PDI of DON from urinary DON biomarker level using Equation (2). For the 54 participants who had urinary total DON higher than LOD, the average PDI was calculated as 13.3 ± 9.7 ng/kg b.w./day, with a maximum of 59.8 ng/kg b.w./day.

### 2.5. Factors that Influence Urinary AFM_1_/DON Levels

There was no statistically significant correlation between urinary total DON level and age or between the male and female groups. Significantly (*p* < 0.001) higher urinary AFM_1_ levels were observed for males compared to those for females (0.029 vs. 0.015 ng/mL). As described above, geographical location is another key factor influencing the urinary AFM_1_ levels (Chak-46 > BP > Chak-48 > Check 49 > KA > BB). Age groups were separated according to the definition by WHO: 2–10 years as children, 10–19 years as adolescents, 19–65 years as adults, and >65 years as elderly. No significant difference in urinary AFM_1_ levels was observed between age groups or between occupation categories (data not shown). 

### 2.6. Association of Urinary AFM_1_/DON Levels with Food Intake

**Figure 1:**
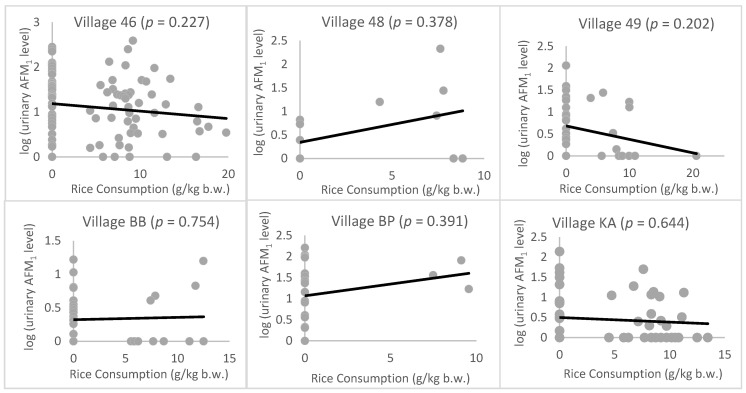
Correlation graphs for urinary AFM_1_ level and rice consumption in each village. Urinary AFM_1_ levels have been log-transformed to normalize the data and rice consumption has been standardized by body weight.

**Figure 2:**
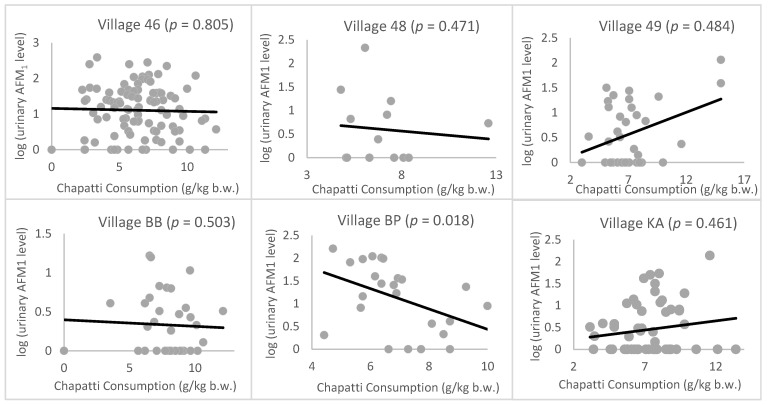
Correlation graphs for urinary AFM_1_ level and chapatti consumption in each village. Urinary AFM_1_ levels have been log-transformed to normalize the data and chapatti consumption has been standardized by body weight.

The association between urinary DON level and food intake was not assessed as none of the food samples tested here showed detectable levels of DON. The correlation between urinary AFM_1_ level and rice/wheat consumption is shown in Figure 1; Figure 2, respectively. There was no significant correlation between wheat consumption or rice consumption and urinary AFM_1_ levels in most villages or in the cohort as a whole. In village BP, a negative correlation between chapatti consumption and urinary AFM_1_ level was observed (*p* = 0.02*). Dietary exposure to AFB_1_ and DON were also calculated by combining the AFB_1_/DON analysis data with the food consumption data. However, there was no significant correlation between the derived dietary exposure level of AFB_1_ or DON and the urinary AFM_1_ or DON levels. 

**Figure 3:**
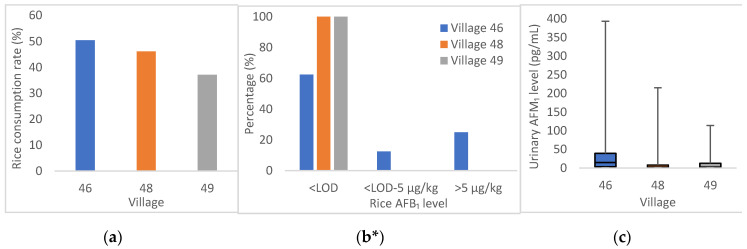
Variation patterns of (**a**) rice consumption rate, (**b**) distribution of AFB_1_ level in rice samples, and (**c**) urinary AFM_1_ among Chak-46, 48, 49 in Sahiwal district. * EU regulation limit for AFB_1_ in rice is 5 µg/kg [24].

The data in Table 4 suggest that rice was one of the major sources of AFB_1_ exposure for the studied population. Figure 3 shows the variation in patterns of rice consumption rate, distribution of AFB_1_ level in rice samples, and urinary AFM_1_ level in the three villages from the Sahiwal district. From Figure 3, village Chak-46 has been observed to have the highest rice consumption (50.5%) among the three villages from Sahiwal district as well as the highest rice AFB_1_ contamination level and urinary AFM_1_ level. A clear pattern can be observed that within the same district, where the villages are geographically close to each other, less rice consumption and lower AFB_1_ contamination in rice can lead to lower urinary AFM_1_ levels. If the data are compared between different districts, where the villages are geographically much further apart, this pattern was not seen. However, participants from Sahiwal district and Kasur district (located in the central Punjab Province) did show a significantly higher urinary AFM_1_ level than participants from Bahawalpur district and Rahim Yar Khan district (located in the south of the Punjab Province).

### 2.7. Risk Evaluation for AFB_1_ and DON Exposure

As AFB_1_ is a Group I carcinogen associated with liver cancer, its exposure can be assessed either by margin of exposure (MoE) or cancer potency. The average MoE of the studied population for aflatoxin was estimated to be 112.9 and 13.2 from Equation (3), using the estimated EDI and PDI, respectively. In addition to MoE, quantitative liver cancer risk assessment was also used to assess the AFB_1_ dietary exposure risk. Considering the synergetic effect of AFB_1_ and hepatitis B virus (HBV) in liver cancer, the carcinogenic potency was estimated separately for HBV positive and HBV negative populations [25,26]. Therefore, the cancer risk related to AFB_1_ exposure is estimated using Equation (4) [17,27,28]. The estimated EDI and PDI were used to calculate the cancer risk, which resulted in AFB_1_-induced cancer risks of 0.059 and 0.514/10^5^ individuals/year, respectively.

The maximum PDI derived from the urine samples was 59.8 ng/kg b.w./day, which is much lower than the PMTDI.

## 3. Discussion

### 3.1. Demographic Characteristics and Correlation with Urinary Biomarkers

Food and urine samples were collected from six different villages located in the Punjab province to assess the AFB_1_ and DON exposure in a rural population from Pakistan. This rural population is characterized by the high proportion (70%) of participants being farmers or involved in farming practice. Participants from village 48 showed significantly higher age compare to those in other villages (apart from village 49), which is potentially caused by the limited sample size. No significant difference was observed for age between villages (*p*-value ranged from 0.084 to 1). Efforts were made to balance the number of participants from both genders. Overall, the number of participants in both genders were similar (58% males); however, due to sample availability, the number of participants from both genders were not balanced in each village. 

Males had significantly higher urinary AFM_1_ levels than females did (0.023 ± 0.056 vs. 0.015 ± 0.034 ng/mL). Several previous papers have reported similar observations for aflatoxin biomarker levels (either in urine or blood) [29,30,31,32]. The underlying mechanism is still not well studied but may involve the gender difference in AFB_1_ biotransformation, which is governed by cytochrome P450 enzymes [33]. The cohort was separated into four different age groups, but no significant difference was observed between these, as seen previously [34,35,36].

### 3.2. Exposure Levels of DON and AFB_1_

Exposure assessment was conducted using validated biomarkers including urinary total DON and AFM_1_ for DON and AFB_1_, respectively [19,37]. Low exposure to DON but high exposure to AFB_1_ was found in this study population. The urinary DON biomarker was detectable in only 20% of samples, whilst the biomarker median level was below the LOD (0.25 ng/mL). This is much lower than that in many other countries previously reported such as U.K. (99%, 7.5 ng/mL) [19]; China (100%, 32.5 ng/mL) [38]; and South Africa (100%, 20.4 ng/mL) [39]. 

In contrast to DON, urinary AFM_1_ was detected in over two-thirds of the samples with a mean level of 0.023 ± 0.048 ng/mL and the maximum reaching up to 0.393 ng/mL. This is comparable to the countries where aflatoxin was considered to be a major health threat, such as Nigeria (72.5%, 0.04 ng/mL) [40] and Tanzania (86%, 0.037 ng/mL) [37], and China in 1988 (54%, 0.048 ng/mL) [41], a period when liver cancer rates were high. Higher average levels of urinary AFM_1_ have also been reported among adults in Ghana in 2006 (mean 1.8, maximum 11.562 pg/mg creatinine); children from Cameroon in 2013 (mean 0.33, maximum 4.7 ng/mL); and children, adolescents, and adults from Nigeria in 2014 (mean 0.3, maximum 1.5 ng/mL) [34,42,43]. 

Among the areas where urinary AFM_1_ data are available, Bangladesh is geographically the closest country to Pakistan and shares some similarities in population ethnicity and dietary habits. A study using high-pressure liquid chromatography with fluorescence detector (LOD 0.0017 ng/mL) conducted in 2017 with 62 participants reported that 40% of the urine samples showed a detectable level of AFM_1_ with an average level of 0.014 ± 0.021 ng/mL, in summer; and an average level of 0.028 ± 0.043 ng/mL in winter [35]. Another study conducted in 2015 involving 95 participants reported 46% of the total urine samples with detectable levels of AFM_1_, with a mean level of 0.08 ± 0.06 ng/mL ranging from 0.031 to 0.348 ng/mL (median 0.061 ng/mL) [44].

Climate is a likely factor explaining the difference in AFB_1_ and DON exposure in the current study population. The climate during the summer in Pakistan is hot, with temperatures ranging from 30 to 48 °C [45]. *Aspergillus* fungi would be able to produce AFB_1_ within this temperature range (maximum mycotoxin production temperature 40 °C), however the temperature is not suitable for the growth of *Fusarium* fungi (maximum mycotoxin production temperature 30 °C), which produces DON [46,47]. High levels of DON have been detected in wheat and corn products from the same studied region in a recent study [18]. More than 40% of samples were tested and found to be contaminated by DON with an average contamination level of 1000 µg/kg, which is close to the EU regulatory limit of 1250 µg/kg. The differences in DON contamination level in food are likely to be caused by changes in farming practice and climate over the year as the samples were collected in 2014 for the present study, whereas the samples were collected during 2017–2018 for the recent study.

### 3.3. Correlation between Urinary AFM_1_ Level and Food Consumption

No significant correlation was observed between wheat consumption and urinary AFM_1_ levels of the studied cohort apart from for village BP, where a negative correlation was observed between wheat consumption and urinary AFM_1_ levels, as shown in Figure 2. Since chapatti is made from wheat, which showed little contamination with AFB_1_, chapatti consumption reduces AFB_1_ exposure. There was a clear correlation between rice consumption, AFB_1_ contamination level in rice, and AFM_1_ levels in the population of three villages from the same region (Figure 3). However, this was not seen in other more geographically separated villages (i.e., village KA, BB, and BP). This may be because in these villages not only rice but other food items were consumed which may also be the probable source of AFB_1_ as found in studies conducted in Brazil and Bangladesh reporting a similar pattern [35,48]. In Pakistan, food commodities including cereals, spices, black tea, and milk have also been reported to be contaminated by aflatoxins to various degrees [21,22,49,50,51], and based on 24 h food dietary data these are likely to contribute to exposure for this cohort [52]. This can also be accountable for the higher PDI estimated from the urinary biomarker compared to EDI estimated by the rice consumption/contamination data. Furthermore, urine samples from the two districts (SW, BP) located in the north of the Punjab province had higher levels of AFM_1_ than the two districts (BB, KA) in the south of the Punjab reflecting comparatively higher exposure of AFB_1_ and agree with the contamination pattern for AFB_1_ in rice collected in these regions from a previous study [17].

A limitation of this study is that few of the rice samples were produced locally in the studied area, and the small number of rice samples collected from the local shops or some of the households may not be representative for the whole studied cohort.

### 3.4. Risk Assessment of DON and AFB_1_

The maximum PDI for DON in this study was 59.8 ng/kg b.w./day, which is 16 times lower than the PMTDI (1000 ng/kg b.w./day). Therefore, based on the low positive rate of participants with detectable level of DON in their urine and low PDI of DON derived from urinary DON level, the studied cohort is under a low risk of DON exposure. 

AFB_1_ has been classified as Class I carcinogen, therefore, human exposure to AFB_1_ should be minimized, and there is no TDI tolerable daily intake for AFB_1_. Here, 69% of the participants showed detectable levels of AFM_1_ in their urine samples with a maximum of 0.393 ng/mL (Table 4). As suggested by the European Food Safety Authority (EFSA), for a substance that is both carcinogenic and genotoxic, such as aflatoxin, an MoE lower than 10,000 should be considered to represent a risk to human health and will require risk-management action [53]. The MoEs derived from either EDI (112.9) or PDI (13.2) were both well below the safe MoE, raising concern for public health from aflatoxin exposure.

Several studies have also evaluated the correlation between AFB_1_ exposure and liver cancer using urinary AFM_1_ level as the biomarker for AFB_1_ exposure. The intervention study conducted in Qidong, China showed a significant declining trend of liver cancer incidences together with a reduction in urinary AFM_1_ level [41]. Over twelve years, the urinary AFM_1_ level reduced from a mean level of 48 with range of 0.006–0.243 ng/mL (54% positive) down to 0.009 ng/mL (only 1 positive sample), correlating with a decrease in liver cancer incidence. The detected level of AFM_1_ in this study is comparable to the level of AFM_1_ in the Chinese study, which suggests relatively high risk of AFB_1_ exposure for this Pakistani population. Significant higher urinary AFM_1_ (0.023 ng/mL) was also observed for patients with liver cancer compared to that of the control group for the study conducted in Taiwan, 2009, and the observed urinary AFM_1_ is also very close to that of the present study [54]. 

Considering the limitation in the estimation of EDI, an AFB_1_-induced cancer risk of 0.514 0.514/10^5^ individuals/year estimated using PDI is discussed here. Compared to the age standard rate for liver cancer in Pakistan, (7.6/10^5^ individuals/year for males and 2.8/10^5^ individuals/year for females), the contribution of AFB_1_-induced cancer risk is found to be significant [55]. In addition, the current cancer risk assessment only assessed the synergetic effect of AFB_1_ and HBV, but recent reports have shown the synergetic effect of AFB_1_ and hepatitis C virus (HCV) in increasing the liver cancer risk [56]. Although the current available research data are not sufficient to conduct cancer risk estimation based on AFB_1_ and HCV infection, the higher prevalence of HCV compared to that of HBV (5.5% vs. 2.4%) in the studied location may further increase cancer risk caused by AFB_1_ exposure [57]. The cancer risk estimation in the current study (0.514 cancer/year/10^5^ individuals) was comparatively higher than the cancer risk reported by Majeed et al. (2018) on the basis of AFB_1_ exposure from rice only in the same location (mean: 0.07 and 0.122 cancer/year/10^5^ individuals in the south and north Punjab province, respectively). This suggests potential AFB_1_ exposure from other dietary sources. Globally, the estimated cancer risk related to AFB_1_ exposure in the present study is much higher than the data reported from China (0.026 cancer/year/10^5^ individuals [28]), where rice is also a staple food, but still lower than that in countries reported to be severely affected by AFB_1_ exposure such as Nigeria (163 cancer/year/10^5^ individuals [27]).

## 4. Conclusions

In this biomarker study of AFB_1_ and DON exposure in a rural population in Pakistan, the results showed a low risk of DON exposure but a high risk of AFB_1_ exposure. The high levels of urinary AFM_1_ were close to levels reported for populations in high liver cancer risk regions. The average biomarker levels varied between different villages and regions, indicating the necessity for epidemiological studies to understand the causes of different exposure levels and the related health consequences. This study revealed the high risk of AFB_1_ exposure in this part of Pakistan, a finding of concern for public health, especially as there is a lack of regulatory enforcement of aflatoxin in Pakistan [58]. 

## 5. Materials and Methods 

### 5.1. Study Population and Sampling

**Figure 4:**
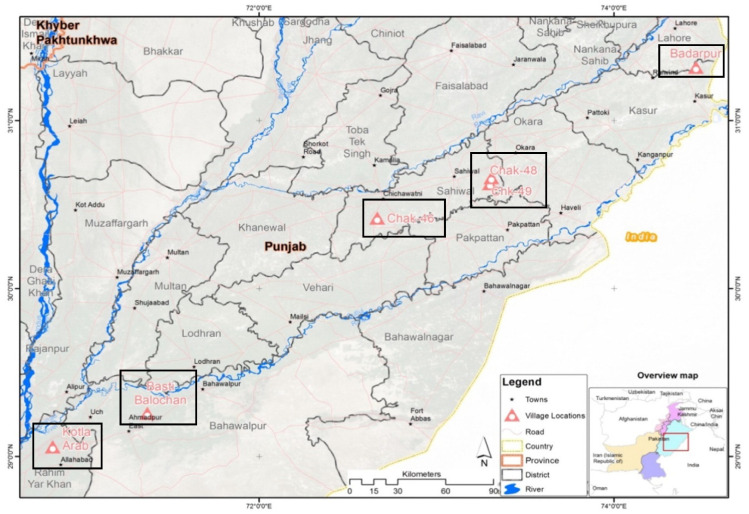
Geographic location of the studied cohort [52].

The study was an extended project of a previous study for which 395 participants were recruited from six villages of four districts of Pakistan for species-based arsenic risk assessment [52]. These villages as shown in Figure 4 included Chak-46, 48, and 49 from district Sahiwal (SW); Badarpur (BP) from district Kasur, Basti Balochan (BB) from Bahawalpur, and Kotla Arab (KA) from Rahim Yar Khan district in the Punjab province, Pakistan.

Food and urine samples were collected in May–July 2014. The staple food consumption such as wheat and rice consumption data on the previous day were obtained using a 24 h dietary recall questionnaire method. Wheat samples (*n* = 195) were provided by some of the households in the study. Few households grew rice, whilst most households purchased rice from local shops or main city markets for their consumption. Following this, a small number of raw rice samples (*n* = 105) were either provided by the household or collected from the local shop. For exposure assessment, spot urine samples (*n* = 301) were collected. All the collected wheat, rice, and urine samples packaged with dry ice were shipped to University of Leeds where these were stored at −20 °C until analysis of AFB_1_, DON, and their urinary biomarkers. All wheat samples had sufficient quantity (>3 g) for analysis of AFB_1_ and DON, whereas only 62 out of the 105 rice samples were available for the same analysis. Of the 301 collected urine samples, only 264 samples had enough volume for the analysis of urinary biomarkers. Of the analyzed urine samples, 58% were from males. 

Informed consent was obtained from all participants. Ethical approval was granted from the University of Leeds Ethical Committee (MEEC 17-036) and the National Bioethics Committee Pakistan (4-87/14/NBC-150/RDC/3).

### 5.2. Food AFB _1_ and DON Extraction

Briefly, 2 g of raw rice or wheat samples were extracted using 8 mL of the extraction solution (79% acetonitrile, 20% water with 1% acetic acid) with 2 h of shaking under 2500 rpm. After the extraction, the mixture was centrifuged (4 °C, 5000× *g*) for 20 min to separate the solid contents. Next, 125 µL of the supernatant was diluted using 875 µL of LC-MS-grade water and then filtered through a 0.22 µm PTFE syringe filter.

### 5.3. Urinary AFB_1_ Biomarker Extraction

Urine samples were first thawed at room temperature and then centrifuged (4 °C, 5000× *g*) to remove impurities. The AFM_1_ was extracted using an AFM_1_ HPLC Immunoaffinity column (IAC, Biocheck, Ascot, UK). 2.5 mL of urine samples diluted in 2.5 mL phosphate-buffered saline (PBS) were loaded, the col-umn washed, and AFM_1_ eluted with 4 mL of methanol. The flow speed was kept at 1 drop/second. Following overnight drying in Gene-Vac E-2 plus (SP scientific, Suffolk, UK), the sample was reconstituted in 250 μL of acetonitrile in water (20%). 

### 5.4. Urinary DON Biomarker Extraction

The major DON metabolites in urine are DON-3-glucuronide (DON-3-GlcA) and DON-15-glucuronide (DON-15-GlcA), with de-epoxy deoxynivalenol (DOM-1) a minor proportion (<10%). To assess the urinary DON biomarker, the urine underwent overnight digestion with β-glucuronidase. After enzymatic digestion, all the glucuronide-conjugated DON species were converted to DON, therefore, the measured DON level would be the sum of free DON and conjugated DON. 

The extraction method was used with minor modifications [19]. In brief, 1 mL of the centrifuged urine sample was spiked with 50 µL of 160 ng/mL internal standard ^13^C_15_-DON, before dilution with 1 mL of PBS, and pH adjusted to 6.8. After overnight digestion at 37 °C using 5750 units of β-glucuronidase, the samples were mixed with 3 mL of PBS for the clean-up by the DONTest^TM^ IAC (Biocheck, Ascot, UK). Following pre-conditioning, the samples were loaded onto the DONTest^TM^ column and let drip through the columns under gravity. Following a washing step, DON was eluted in 4 mL methanol. Samples were dried overnight before being reconstituted in 250 µL of methanol: water (10: 90).

### 5.5. LC-MS/MS Analysis

Rice/wheat AFB_1_ and DON level as well as urinary AFM_1_ and total DON level was measured by a Thermo Vanquish Flex binary ultra-performance liquid chromatography (UPLC) system coupled with Thermo TSQ Quantiva triple Quadrupole mass spectrometer (MS) using an electrospray ionization source (ESI) (Thermo, Manchester, UK). The extracted urine samples (5 µL) were injected on a Waters Acquity UPLC ethylene bridged hybrid (BEH) C18 column (2.1 × 50 mm, 1.7 µm particle size) operated at 40 °C. The solvent gradient began with 90% mobile phase (A) 0.1% formic acid in water and 10% mobile phase (B) methanol; reached 100% B over 15 min; the system was retained at 100% B for 3 min, followed by10% B to equilibrate the system for 2 min. The flow rate was 0.35 mL/min. 

The MS was operating in positive mode with 3.35 kV spray voltage, 50 arb sheath gas, 15 arb auxiliary gas, 2 arb sweep gas, the ion transfer tube was set at a temperature of 350 °C, and the vaporization temperature was 400 °C. The AFB_1_, AFM_1_, and DON levels were quantified in the multiple reaction monitoring (MRM) mode. For AFB_1_, the molecular ion scan was set at m/z 313 ([M + H]^+^) and product ion scan at m/z 285 (quantification ion) and m/z 269 (qualification ion). For AFM_1_, the molecular ion scan was set at m/z 329 ([M + H]^+^) and product ion scan at m/z 273 (quantification ion) and m/z 229 (qualification ion). For DON, the molecular ion scan was set at m/z 297.08 ([M + H]^+^) and product ion scan at m/z 203 (quantification ion) and m/z 231 (qualification ion). For the ^13^C_15_-DON internal standard the molecular ion scan was set at m/z 312.17 ([M + H]^+^) and product ion scan at m/z 263 and m/z 186. The concentration of all the analytes were quantified using the Quan Browser built in Xcalibur 4.1 software. The rice/wheat DON, AFB_1_, and urinary AFM_1_ level were determined by comparing the peak area to an external standard curve, whereas urinary DON level was corrected by the signal of the internal standard.

Wheat/rice AFB_1_ and urinary AFM_1_ levels were quantified by a calibration curve established by pure standards in 20% acetonitrile in water. The calibration curve showed linearity (R^2^) of 0.998 within the range of 0.005–2 ng/mL. Wheat/rice DON levels were quantified by a calibration curve established by pure standards in 10% methanol in water, whereas urinary DON levels were quantified by a calibration curve based on the ratio of the signal of the pure standards with internal standard in 10% methanol in water. The linearity of the DON calibration curves (R^2^) was 0.999 within the range of 2–250 ng/mL. A signal to noise ratio (S/N) of 3 and 10 was used to determine the limit of detection (LOD) and limit of quantification (LOQ), respectively, all the LOD and LOQs were calculated considering the dilution/concentration factors during the extraction step. The LOD and LOQ for rice/wheat AFB_1_ analysis were 0.064 and 0.160 µg/kg, whereas the LOD and LOQ for rice/wheat DON analysis were 32 and 64 µg/kg, those for urinary AFM_1_ analysis were 0.002 and 0.005 ng/mL, whereas the LOD and LOQ for urinary DON were 0.25 and 0.5 ng/mL.

### 5.6. Daily Intake Estimation for AFB_1_ and DON

Either dietary intake or biomonitoring approaches can be used to estimate the daily intake of mycotoxins as EDI or PDI, respectively. For EDI, it can be calculated using Equation (1). The food consumption data for rice and chapatti are available from the 24 h dietary recall questionnaire. However, as none of the rice or wheat samples showed a detectable level of DON, EDI can only be derived for AFB_1_.

(1) $$\mathrm{EDI}\;=\frac{\;\mathrm{Food}\;\mathrm{consumption}\;\left(\mathrm{kg}/\mathrm{day}\right)\times \mathrm{Food}\;\mathrm{contamination}\;\mathrm{level}\;\left(µg/\mathrm{kg}\right)}{\mathrm{body}\;\mathrm{weight}\;\left(\mathrm{kg}\right)}$$

PDI can be calculated using Equation (2). Individual body weight was available, but the daily urine volume was not available in the study. Daily urine volumes were assumed to be 36 and 18 mL/kg/day for children (4–10 years) and adolescents (10–19 years), and 2000 mL/day for adult males and 1600 mL/day for adult females, respectively, as recommended by EFSA (2012). The absorption, distribution, metabolism, and excretion of DON in the human body is relatively well studied. According to the European Food Safety Authority (EFSA), the DON excretion rate is estimated to be 70% [59]. According to Zhu et al., the AFB_1_ excretion rate was estimated to be 2% [60].

(2) $$\mathrm{PDI}=\;\frac{\mathrm{Urinary}\;\mathrm{biomarker}\;\mathrm{level}\left(\mathrm{ng}/\mathrm{mL}\right)\times \mathrm{volume}\;\mathrm{of}\;\mathrm{daily}\;\mathrm{urine}\;\mathrm{excretion}\;(\mathrm{mL}/\mathrm{day})}{\mathrm{Body}\;\mathrm{weight}\left(\mathrm{kg}\;b.w.\right)\;\times \;\%\;\mathrm{biomarker}\;\mathrm{urine}\;\mathrm{excretion}\;\mathrm{rate}}$$

### 5.7. Risk Assessment for AFB_1_ and DON

As AFB_1_ is a genotoxic carcinogen, the risk of AFB_1_ exposure is evaluated by margin of exposure (MoE), which can be calculated using Equation (3):

(3) $$\mathrm{MoE}=\;\frac{\mathrm{Benchmark}\;\mathrm{dose}\;\mathrm{lower}\;\mathrm{confidence}\;\mathrm{limit}\;\mathrm{of}\;10\%\;\mathrm{extra}\;\mathrm{risk}\left({\mathrm{BMDL}}_{10}\right)}{\mathrm{EDI}\;\mathrm{or}\;\mathrm{PDI}}$$

where the BMDL_10_ was suggested to be 400 ng/kg b.w./day by EFSA for aflatoxin [4].

Cancer potency can also be used to quantitatively assess the exposure risk to AFB_1_ by using Equation (4), where P_cancer_ calculated using Equation (5) is the cancer potency, which is considered as the potency of AFB_1_-induced cancer for both hepatitis B surface antigen positive (HBsAg+) and hepatitis B surface antigen negative (HBsAg−) individuals. The carcinogenic potency of AFB_1_ for non-HBV carriers (P_HBsAg−_) is 0.01 cancer/year/ng AFB_1_/kg b.w./day per 10^5^ individuals [61,62]. For HBV carriers, the carcinogenic efficiency of AFB_1_ is 30 times higher than that for non-carriers; therefore, the carcinogenic potency of AFB_1_ for HBV carriers (P_HBsAg+_) is 0.3 cancer/year/ng AFB_1_/kg b.w./day/10^5^ individuals [26]. The infection rate of HBV in Pakistan is 2.4% [63], which means %Pop.HBsAg^+^ is 2.4% and %Pop.HBsAg^−^ is 97.6%.

(4) $$Cancer\;risk=P_{cancer}\;\times \mathrm{EDI}\;\left(\mathrm{or}\;\mathrm{PDI}\right)$$

(5) $$P_{cancer}=(P_{HBsAg+}\times \%Pop.HBsAg^{+})+(P_{HBsAg-}\;\times \%Pop.HBsAg^{-})$$

The risk of DON exposure is evaluated by comparing the PDI level to the PMTDI level (1 μg/kg b.w. per day).

### 5.8. Statistical Analysis

Means (± SD), medians, and range were used to summarize the levels of the mycotoxin biomarkers. For levels below the LOD, a value of 1/2 LOD was assigned for statistical calculation [37]. Linear correlation and regression analysis were used to investigate the correlation between urinary AFM_1_ and DON level and food consumption data. Wheat and rice consumption data were adjusted by participants’ body weight. Overall, 29% of the rice consumption was considered as raw rice. Nonparametric tests were used to evaluate the correlation between the urinary mycotoxin biomarker levels and the demographic data (Mann–Whitney test or Kruskal–Wallis test depending on the number of groups). All analyses were carried out using IBM SPSS Statistics Version 25 and a *p*-value of less than 0.05 was used to assign for statistical significance.

## Figures and Tables

**Table 1:** The reliability and variation of method assessed using quality control samples (*n* = 15) for both urinary AFM_1_ (hydroxylated form of AFB_1_) and deoxynivalenol (DON) measurement.

AFM_1_			DON		
Spiked Level (ng/mL)	Recovery Mean ± SD (%)	CV (%)	Spiked Level (ng/mL)	Recovery Mean ± SD (%)	CV (%)
0.15	116 ± 18.5	16	8	91 ± 11.7	13
1	98 ± 10.4	11	25	98 ± 8.6	9
15	85 ± 11.9	14	125	103 ± 9.5	9

Blank urine samples were spiked at three different levels and extracted together with each batch of urine samples. In total, 15 sets of quality controls (QCs) were extracted and analyzed. CV—coefficient of variance.

**Table 2:** Demographics of the participants in the six sampled villages.

Characteristics	Chak-46	Chak-48	Chak-49	BB	BP	KA	All Villages
Total n	99	13	35	35	24	58	264
Male n, (%)	63 (64)	13 (100)	29 (83)	11 (31)	15 (63)	22 (38)	153 (58)
Age							
Mean ± SD (range)	32.1 ± 17.7 (4–75)	42.5 ± 12.1 (17–61)	41.4 ± 17.3 (6–70)	36.1 ± 17.0(9–65)	37.6 ± 16.5 (15–65)	32.9 ± 16.7(9–80)	35.0 ± 17.5 (4–80)
Occupation *n* (%)							
Farmer	58 (59)	11 (85)	29 (83)	25 (71)	21 (88)	39 (67)	185 (70)
Student	27 (27)	1 (7.5)	5 (14)	6 (17)	3 (12)	14 (24)	56 (21)
Other	14 (14)	1 (7.5)	1 (3)	4 (11)	0 (0)	4 (9)	24 (9)
Chapatti * consumption (g/kg b.w./day)							
Mean ± SD	6.4 ± 2.3 (0–12.1)	7.0 ± 2.1 (4.8–12.6)	7.2 ± 2.6 (3.0–15.0)	7.8 ± 2.1 (0–12.1)	6.9 ± 1.4 (4.4–10.0)	7.3 ± 2.0 (3.2–13.4)	7.0 + 2.2 (0–15.0)
Rice consumption (g/kg b.w./day)							
Consumption rate (%)	50.5	46.2	37.1	34.3	12.5	44.8	41.7
Mean ± SD (range)	9.6 ± 3.6 (4.3–19.8)	7.4 ± 1.6 (4.3–8.8)	9.1 ± 4.0 (3.9–20.6)	8.8 ± 2.7 (5.5–12.5)	8.7 ± 1.1 (7.5–9.6)	8.8 ± 2.2 (4.5–13.5)	9.1 ± 3.1 (3.9–20.6)

* Chapatti: one of the staple foods in Pakistan, made from wheat flour. BB—Basti Balochan; BP—Badarpur; KA—Kotla Arab.

**Table 3:** Occurrence and level of aflatoxin B_1_ (AFB_1_) in the rice/wheat samples collected from the cohort.

Villages	Chak-46	Chak-48	Chak-49	BB	BP	KA	All Villages
Rice ^*^ (*p* = 0.04)							
Positive *n* ^a^ (%)	3/8 (38)	0/1 (0)	0/5(0)	13/17 (76)	9/15 (60)	16/16 (100)	41/62 (66)
Mean ± SD (µg/kg)	5.65 ± 9.51	nd	nd	10.17 ± 18.02	5.11 ± 11.94	1.09 ± 1.40	5.04 ± 11.94
Median (range) (µg/kg)	0.03 (nd–23.58)	nd (nd)	nd (nd)	2.46 (nd–71.56)	0.37 (nd–36.88)	0.42 (0.2–5.26)	0.38 (nd–71.56)
Wheat (*p* = 0.93)							
Positive *n* (%)	1/40 (3)	1/45 (2)	1/51 (2)	0/13 (0)	0/17 (0)	0/29 (0)	3/195 (2)
Mean ± SD (µg/kg)	0.04 ± 0.03	0.03 ± 0.06	0.06 ± 0.22	nd	nd	nd	0.04 ± 0.12
Median (range) (µg/kg)	nd (nd–0.23)	nd (nd–0.42)	nd (nd–1.59)	nd (nd)	nd (nd)	nd (nd)	nd (nd–1.59)

^a^ Rice was grown locally only to a limited extent and only few households produced rice, which they keep for personal consumption and sell out the excessive quantity, whilst most households purchase from local shops or main city markets. nd: non-detectable value; *p* values: * for *p* < 0.05, statistical significance compared with villages for urinary biomarker levels using *K* independent sample nonparametric test.

**Table 4:** Occurrence and level of urinary DON and AFM_1_ of the cohort.

Villages	Chak-46	Chak-48	Chak-49	BB	BP	KA	All Villages
DON (*p* = 0.46)							
Positive *n* (%)	18/99 (18)	0/13 (0)	8/35 (23)	11/35 (31)	7/24 (29)	10/58 (17)	54/264 (20)
Mean ± SD (ng/mL)	0.166 ± 0.113	nd	0.156 ± 0.064	0.202 ± 0.187	0.169 ± 0.080	0.174 ± 0.165	0.170 ± 0.129
Median (range) (ng/mL)	nd (nd–0.901)	nd (nd)	nd (nd–0.348)	nd (nd–1.177)	nd (nd–0.388)	nd (nd–1.247)	nd (nd–1.247)
AFM_1_^***^ (*p* < 0.001)							
Positive *n* (%)	86/99 (87)	7/13 (54)	21/35 (60)	19/35 (54)	20/24 (83)	29/58 (50)	182/264 (69)
Mean ± SD (ng/mL)	0.039 ± 0.015	0.022 ± 0.059	0.011 ± 0.021	0.003 ± 0.004	0.037 ± 0.045	0.009 ± 0.021	0.023 ± 0.048
Median (range) (ng/mL)	0.015 (nd–0.393)	0.003 (nd–0.215)	0.003 (nd–0.114)	0.002 (nd–0.017)	0.020 (nd–0.162)	0.001 (nd–0.137)	0.004 (nd–0.393)

nd: non-detectable value; *p* values: *** for *p* < 0.001, statistical significance compared with villages for urinary biomarker levels using K independent sample nonparametric test.
